# Phytoconstituents, Antioxidant Activity and Cytotoxicity of *Puya chilensis* Mol. Extracts in Colon Cell Lines

**DOI:** 10.3390/plants13212989

**Published:** 2024-10-26

**Authors:** Manuel Martínez-Lobos, Valentina Silva, Joan Villena, Carlos Jara-Gutiérrez, Waleska E. Vera Quezada, Iván Montenegro, Alejandro Madrid

**Affiliations:** 1 Laboratorio de Productos Naturales y Síntesis Orgánica (LPNSO), Departamento de Ciencias y Geografía, Facultad de Ciencias Naturales y Exactas, Universidad de Playa Ancha, Avda. Leopoldo Carvallo 270, Playa Ancha, Valparaíso 2340000, Chile; manuel.martinez@upla.cl (M.M.-L.); silvapedrerosv@gmail.com (V.S.); 2 Centro Interdisciplinario de Investigación Biomédica e Ingeniería para la Salud (MEDING), Escuela de Medicina, Facultad de Medicina, Universidad de Valparaíso, Valparaíso 2340000, Chile; juan.villena@uv.cl; 3 Centro Interdisciplinario de Investigación Biomédica e Ingeniería para la Salud (MEDING), Escuela de Medicina, Escuela de Kinesiología, Universidad de Valparaíso, Valparaíso 2340000, Chile; carlos.jara@uv.cl; 4 Laboratorio de Química de Metabolitos Bioactivos, Escuela de Química y Farmacia, Facultad de Farmacia, Centro de Investigación Farmacopea Chilena, Universidad de Valparaíso, Valparaíso 2340000, Chile; waleska.vera@uv.cl; 5 Centro Interdisciplinario de Investigación Biomédica e Ingeniería para la Salud (MEDING), Escuela de Obstetricia, Facultad de Medicina, Universidad de Valparaíso, Valparaíso 2340000, Chile; ivan.montenegro@uv.cl

**Keywords:** *Puya chilensis*, antioxidant, cytotoxic, colon cancer, ascorbic acid 2,6 dihexadecanoate

## Abstract

*Puya chilensis* Mol. is a plant of the Bromeliaceae family, which has been traditionally used for medicinal applications in various digestive disorders. In this study, the phytoconstituents of six extracts of stems and flowers of *P. chilensis* were evaluated: phenols, flavonoids and total anthraquinones, as well as their antioxidant capacity and cytotoxicity in colon cancer cell lines HT-29. The data demonstrate that the ethyl acetate extract of *P. chilensis* flowers is cytotoxic in HT-29 cell lines (IC_50_ = 41.70 µg/mL) without causing toxic effects on healthy colon cells (IC_50_ > 100 µg/mL); also, this extract concentrated the highest amount of phenols (4.63 μg GAE/g d.e.), flavonoids (31.5 μg QE/g d.e.) and anthraquinones (12.60 μg EE/g d.e.) among all the extracts tested, which also correlated with its highlighted antioxidant capacity (DPPH∙IC_50_ = 4.15 mg/mL and FRAP 26.52 mM TEAC) over the other extracts. About thirty-five compounds were identified in this extract−the fatty acid esters present have been shown to have therapeutic effects on several types of cancer and could explain its antiproliferative activity.

## 1. Introduction

Cancer is the second leading cause of human death worldwide. Among the different types of human cancer, colorectal cancer is the second deadliest and it is estimated that in 2020 alone there were more than 930,000 deaths from this cause. By 2040, the lethality of colorectal cancer is projected to increase by about 73% [1]. Currently, treatment with surgery, radiation and chemotherapy are limited in terms of tolerance, efficacy and cross-resistance. However, recent research has linked a diet rich in fruit and vegetables to the prevention of colon cancer [2,3]. This benefit is attributed in part to the polyphenols present, which have potent antiproliferative effects on cancer cells [4,5]. In this regard, the Bromeliaceae *Ananas comosus*, known as “pineapple”, has been used medicinally by tropical natives for centuries as a digestive aid and wound healer [6]. Studies have indicated that pineapple juice is capable of inhibiting growth of colon cancer cells [7].

Among the species of Bromeliaceae, the genus *Puya* stands out, which is composed of more than 200 species native to the Andes and Central America [8]. These plants are generally characterized by being monocarpic (the plant dies after producing a flower and seeds) [9]. In Chile, a total of nine species of *Puya* are recognized and the most abundant species is the *Puya chilensis* Mol., known as “chagual”, a name that comes from the Quechua language word “ch’ahuar” or “ch’auwar” that would mean “tow” or “bristle”, which is explained by the ancient extraction of fiber from the leaves to make twine and yarn used for fishing nets; its flowers were also used as ornaments in festivities [10]. *P. chilensis* has been used for centuries as a plant for human food, either fresh or processed [11]. In addition, this plant was used in folk medicine as an astringent and moisturizing agent, as an antipyretic, anti-inflammatory and antidiarrheic [12,13,14]. There have also been reports that methanol extracts of *P. chilensis* meristems and leaves possess pharmacological properties, such as antioxidant and α-glucosidase inhibitory activities, and extracts of the stems have been reported to exhibit anticancer activity on human hepatocellular carcinoma [15,16]. Despite the few studies conducted on this plant, there is insufficient information on the antioxidant and cytotoxic properties of *P. chilensis* stems and flowers. Therefore, the aim of this study was to estimate the content of phenols, flavonoids and anthraquinones, and to evaluate and compare the in vitro antioxidant and cytotoxic properties of sequential extracts of increasing polarity of *P. chilensis* stems and flowers in colon cancer (HT-29) and colon non-cancer (CCD 841 CoN) cells.

## 2. Results and Discussion

The plant’s constituents’ extraction with increased polarity solvents hexane (**H**), ethyl acetate (**EA**) and ethanol (**E**), resulted in six extracts of *P. chilensis*, of which three correspond to stem (**S**) extracts and three to flowers (**F**). The yield of **SH**, **SEA**, **SE**, **FH**, **FEA** and **FE** extraction from *P. chilensis* were 0.33%, 0.81%, 3.44%, 0.23, 0.61 and 1.65% respectively. After the extracts were obtained, the total content of phytoconstituents was measured using colorimetric assays, as summarized in Table 1.

The total phenolic content of extracts from *P. chilensis* stems and flowers varied slightly, with the **EA** extract of flowers showing the highest phenolic content of the extracts tested. The presence of flavonoids was recorded in all the extracts except in the **E** extract of the flowers. The non-detection of flavonoids in this extract is probably due to the non-polar nature of flavonoids, such as isoflavones, flavanones, flavones and flavonols, which have an affinity for solvents such as *n*-hexane, chloroform, dichloromethane, diethyl ether and ethyl acetate, which are found in the flower of *P. chilensis* [17,18]. The analysis also showed that the extracts of low and medium polarity from flowers have a higher anthraquinone content, which is to be expected, because an important group of compounds with anthraquinone skeletons are known to be naturally occurring pigments that give a yellow to red coloring to flowers and are often found in extracts of floral origin [19].

The DPPH, FRAP and TRAP assays were used to evaluate the antioxidant activity of *P. chilensis* extracts (see Table 2).

In general, a moderate amount of phytochemicals is correlated with a moderate antioxidant capacity. According to our results presented in Table 2, the highest capacity to neutralize DPPH radicals was found in the **EA** extract of flowers, with an IC_50_ value of 4.15 mg/mL. In turn, the stem **EA** extract and both **H** extracts showed low radical scavenging capacity. On the other hand, both **E** extracts showed low scavenging activity with IC_50_ values of 5.32 and 11.77 mg/mL in flowers and stems, respectively. The FRAP assay represents the electron donating capacity of the samples, thus allowing the determination of their reducing power [20]. The results of the FRAP assay indicated contrasting results for the extracts of both flowers and stems of *P. chilensis* (Table 2). Thus, the highest reducing power among the samples tested was exhibited by the flower **EA** extract with a value of 26.52 mM. However, all samples were more active than the control. Additionally, flower **EA** extract was found to be the most active of all extracts tested in the TRAP assay, however when comparing the active extract activity with the pure controls, it was almost 3 times less potent. This may be explained by the fact that the extract is a mixture of active components, between which antagonism may exist [21].

In the present study, the cytotoxic activity of *P. chilensis* stems and flowers against two human colorectal cell lines was assessed; HT-29 (adenocarcinoma) and CCD 841 CoN (epithelial), using the Sulforhodamine B (SRB) assay using doxorubicin (Doxo) and 5-fluorouracil (5-FU) as control drugs. Results are shown in Table 3.

Cytotoxic activity can be classified according to the median inhibition values (IC_50_) obtained. A literature review in this regard indicated that activity levels can be labeled as potentially cytotoxic (IC_50_ > 100 µg/mL) and highly selective (SI > 3), or moderately cytotoxic (IC_50_ > 100) but less selective (SI < 3) [22,23]. In this sense, the results shown in Table 3 show that **EA** from *P. chilensis* flowers presents potentially cytotoxic activity in the HT-29 cell line with an IC_50_ value of 41.70 µg/mL, effectively correlating the phytochemical content and the antioxidant power shown by this extract, as has been validated in other studies of edible or medicinal plants [24]. Furthermore, its selectivity index is close to 3 and according to the above, would prove to be selective. This extract would have a high to moderate selectivity comparable to doxorubicin, which causes approximately 20 times more damage to normal colon epithelial cells CCD 841 CoN than **EA** flower extract.

The antiproliferative activity of **EA** flower extract from *P. chilensis* could be in part due to the action of the flavonoids present in the extract. Some of these, as well as other phenolic constituents, were reported in the leaves of *P. chilensis* and *P. alpestris* [15,25]. The flavonol quercetin, for example, has several biological properties. In fact, quercetin is a unique compound because of its potential to fight cancer-related diseases in a multi-targeted manner [26]. As well, the flavone apigenin has been shown to have broad anticancer effects in several types of cancer, including colorectal [27]. In addition, the methoxylated trisubstituted flavonol laricitrin can inhibit the growth of epithelial colorectal adenocarcinoma cells [28]. It is noteworthy that all extracts presented IC_50_ values above 100 µg/mL in non-cancerous cells, which allows us to conclude that *P. chilensis* extracts have no adverse effects on healthy colon cells and therefore supports its secure consumption for the ancestral therapeutic effects attributed to this species.

Based on the results obtained, the most active extract was characterized by gas chromatography coupled to mass spectrometry (GC-MS). This technique is commonly used to obtain an effective profile of secondary metabolites present in edible plants, flowers and fruits [29,30], and is also a useful way to determine the volatiles present in extracts of medium polarity [31]. The phytochemical profile of the volatile fraction of **EA** flower extract from *P. chilensis* is presented in Table 4.

Thirty-five compounds were identified in the **EA** flower extract from *P. chilensis*, which represented 82.96% of the volatiles of the **EA** extract. The major constituents of the volatiles of the **EA** flower extract were ascorbic acid 2,6-dihexadecanoate (15.21%), 1-pentacosanol (12.57%), stigmast-4-en-3-one (10.59%), (*Z*,*Z*)-9,12-octadecadienoic acid (10.41%) and 1-tetracosanol (9.85%). Most of the volatile compounds extracted were fatty acid esters, fatty alcohols, fatty acids, steroids and to a lesser extent benzoic acid derivatives; similarly, previous data showed the existence of derivatives of hydroxybenzoic acid in stems of *P. chilensis* [15,16] and other edible plants [33]. In this context, the nature of the metabolites determined can be attributed not only to the polarity of the extraction solvent and genetic diversity of the species [34], but also to the properties of the GC method, characterized to determine volatile compounds [31]. Ascorbic acid esters have demonstrated strong antioxidant, antibacterial and cytotoxic activity [35]; in this sense, the percentage of ascorbic acid 2,6-dihexadecanoate could contribute to the antioxidant and antiproliferative activity of the **EA** flower extract against tumor cells, as has been reported previously [36,37]. Fatty acids and their respective esters constitute a wide range of materials used in the discovery and formulation of active ingredients of pharmaceutical importance, because they have been reported as potential antioxidant and antiproliferative compounds [38,39]. In addition, fatty acid esters have been shown to modulate the anti-inflammatory response of macrophages [40], which would contribute to the antiproliferative capacity of the extract. Likewise, fatty alcohols have shown potent anti-inflammatory activity; among them, 1-tetracosanol has shown antiproliferative effects in human melanoma cell lines [41] and 1-pentacosanol is a potential inhibitor of prostate tumor cell proliferation [42]. Both alcohols could contribute to the activity shown by the **EA** floral extract of *P. chilensis*.

The results obtained in the present study call for further study of *P. chilensis* flowers in ethnopharmacological terms, such as evaluating the antibacterial potential of this plant against pathogens associated with gastric pathologies in order to enhance the therapeutic use of *P. chilensis*, especially as a potent source of active metabolites such as fatty acids, phenolic acids and their respective derivatives, and the possibility for investigators to develop multiple bioactive agents with therapeutic effects against various malignant neoplasms.

## 3. Materials and Methods

### 3.1. Plant Material

Stems and flowers of *P. chilensis* were collected in January 2023 from Federico Santa María cliffs (33°03′04″ S 71°39′34″ O) in Valparaíso, Chile. Botanical identification and authentication were verified by Mr. Patricio Novoa, Forest Engineer, Botanical Expert, and Chief of the Horticulture Department, National Botanic Garden of Viña del Mar, Valparaíso, Chile. Voucher specimen has been deposited in the Herbarium of Natural Products Laboratory of Universidad de Playa Ancha, Valparaíso, Chile (PC-012023).

### 3.2. Preparation of Plant Extract

At the laboratory, the stems and flowers were rinsed with water and air-dried. The stems and flowers were then dried in a food dehydrator at 60 °C for 14 h and 50 °C for 20 h, respectively. Finally, both plant parts were ground in a kitchen grinder to obtain a fine powder. Later, 300 g of dried powder of *P. chilensis* stems and flowers separately was extracted using maceration containers at room temperature for 72 h using **H** and the residue was extracted successively with **EA** and **E**, using 1 L of each solvent in the extraction. All solutions were evaporated and dried under vacuum (below 40 °C), and then each extract was kept in darkness at room temperature.

### 3.3. Analysis of Total Polyphenolic, Total Flavonoid and Anthraquinones Content

According to protocols by Jara et al. [43], total phenolic content was established employing the Folin–Ciocalteu method, total flavonoid content was established employing the Dowd method and total anthraquinone content was determined by the Arvouet-Grand method using a UV/VIS spectrometer, UV-2601 (Ray-LEIGH, Beijing, China). It is important to note that the detection limit (LOD) of the equipment is 0.001 nm for absorbance.

#### 3.3.1. Phenols

Five hundred microliters of the extract solution in ethanol (1.0 mg/mL) was mixed with a Folin–Ciocalteu reagent (2.5 mL, 0.2 N) and incubated for 5 min. Then, a 7.5% *w*/*v* Na_2_CO_3_ solution (2.0 mL) was added and the mix was incubated in the dark at room temperature for 2 h. The absorbance of the solution was measured at 700 nm using ethanol as the blank. The obtained absorbance values were interpolated using a gallic acid standard curve (0–100 mg/L) and the total phenolic content was expressed as µg of gallic acid equivalents (GAE) per g of dried extract (d.e.). Values shown are the mean ± standard deviation of three independent experiments performed in triplicate.

#### 3.3.2. Flavonoids

One milliliter of 2% *w*/*v* aluminum chloride (AlCl_3_) in ethanol was mixed with the same volume of the extract solution in ethanol (1.0 mg/mL). The mix was incubated for 10 min at room temperature, and absorbance was measured at 415 nm against a blank sample consisting of a 1.0 mL extract solution with 1.0 mL of ethanol without AlCl_3_. The absorbance values were interpolated using a quercetin calibrate curve (0–100 mg/L). The total flavonoid content was expressed as μg of quercetin equivalents (QE) per g of dried extract (d.e.). Values shown are the mean ± standard deviation of three independent experiments performed in triplicate.

#### 3.3.3. Anthraquinones

One milliliter of 2% *w*/*v* AlCl_3_ in ethanol was mixed with the same volume of the extract solution in ethanol (1.0 mg/mL). The mix was incubated for 10 min at room temperature, and absorbance was measured at 486 nm against a blank sample consisting of 1.0 mL extract solution with 1.0 mL of ethanol without AlCl_3_. The absorbance values were interpolated using an emodin calibrate curve (0–100 mg/L). The total anthraquinone content was expressed as μg of emodin equivalents (EE) per g of dried extract (d.e.). Values shown are the mean ± standard deviation of three independent experiments performed in triplicate.

### 3.4. GC-MS Identification of Compounds

The **EA** flower extract from *P. chilensis* was analyzed by GC-MS for volatiles and semi-volatiles, using Shimadzu GCMS-QP2010 Plus combination (Shimadzu, Kyoto, Japan) coupled with a fused silica RTX-5 capillary column (30 m × 0.25 mm id, 0.25 um film; Restek, Bellefonte, PA, USA). The protocol and working conditions used were those previously described by Faundes-Gandolfo [44]. The GC was operated in splitless mode (30 s sampling time and 1 μL of sample) with helium as the carrier gas (1 mL min^−1^ flow) and an injector temperature of 200 °C. The oven and the column were programmed from 50 °C (2 min hold) to 280 °C at a rate of 8 °C min^−1^ (15 min hold). The mass spectrum was acquired by electronic impact at 70 eV with a mass range of 35 to 500 *m*/*z* in full scan mode (1.56 scan s^−1^). Compounds in the chromatograms were identified by comparing their mass spectra with those in the NIST20 library. Chromatographic peaks were considered “unknown” when their similarity index (MATCH) and reverse similarity index (RMATCH) were less than 800, and discarded in this identification process [45]. These parameters are referred to by the degree the target spectrum matches the standard spectrum in the NIST Library (the value 1000 indicates a perfect fit), and by comparison of their retention index with data published in other studies for the same type of column. The retention indices were determined under the same operating conditions in relation to a homologous n-alkanes series (C_8_–C_36_) by Equation (1):

**RI** = 100 × (n + Tr(_unknown_) − Tr(_n_)/Tr(_N_) − Tr(_n_)) (1)

where n = the number of carbon atoms in the smaller *n*-alkane; N = the number of carbon atoms in the larger *n*-alkane; and Tr = the retention time. Components’ relative concentrations were obtained by peak area normalization.

### 3.5. DPPH Free Radical Scavenging Assay

*P. chilensis* extracts were tested in vitro using the DPPH (2,2-diphenyl-1-picrylhydrazyl) assay according to the protocol described previously in [46]. The sample (100 μL, extracts at 0–100 mg/mL) was mixed with a 50 μM DPPH solution (2.9 mL) freshly prepared in ethanol. A 50 μM DPPH solution (2.9 mL) with ethanol (0.1 mL) was used as the control. The sample and control solutions were incubated for 15 min at room temperature, and the absorbance was measured at 517 nm. The inhibition (%) was calculated by Equation (2):

**I%** = 100% (A_control_−A_sample_)/A_control_ (2)

From the obtained I% values, the IC_50_ value was determined by linear regression analysis. All the measurements were obtained from three independent experiments, each performed in triplicate.

### 3.6. Ferric Reducing Power (FRAP) Assay

The experiment was performed on the basis of the protocol described by Mellado et al. [46]. Freshly prepared (10 volumes of 300 mM acetate buffer, pH 3.6, with 1.0 volume of 10 mM TPTZ (2,4,6-tri(2-pyridyl)-s-triazine) in 40 mM hydrochloric acid, and 1.0 volume of 20 mM ferric chloride) FRAP reagent (3.0 mL) was mixed with deionized water (300 μL) and the sample (100 μL, 1.0 mg/mL of each extract). The mix was incubated for 30 min at 37 °C in a water bath and the absorbance was measured at 593 nm using ethanol as the blank solution. The obtained absorbance values were interpolated in a Trolox calibrate curve (0–200 mg/L) and the FRAP values were expressed in mM Trolox equivalent antioxidant capacity (mM TEAC). Values shown are the mean ± standard deviation of three independent experiments performed in triplicate.

### 3.7. Total Reactive Antioxidant Power (TRAP) Assay

The experiment was performed following the protocol previously published by Mellado et al. [46]. One volume of 10 mM solution of ABAP (2,2′-azo-bis(2-amidino propane) was mixed with the same volume of 150 μM solution of ABTS (2,2′-Azino-bis(3-ethylbenzthiazoline-6-sulfonic acid) using PBS 100 mM at pH of 7.4 (TRAP solution). The mixture was incubated at 45 °C for 30 min and then cooled to room temperature for use. Sample solution (10 μL, 1.0 mg/mL of each extract) was mixed with the TRAP solution (990 μL), and the absorbance was measured after 50 s at 734 nm against the ABTS solution as the blank. The absorbance values were interpolated in a Trolox standard curve (0–120 mg/L). Values shown are the mean ± standard deviation of three independent experiments performed in triplicate.

### 3.8. In Vitro Cytotoxicity Assay

#### 3.8.1. Cells and Culture Conditions

The cell lines were purchased from the American Type Culture Collection (Rockville, MD, USA): HT-29 (human colon cancer) and CCD 841 CoN (human colon epithelial cells). The cell lines were maintained in a 1:1 mixture of Dulbecco’s modified Eagle’s medium (DMEM) and Ham’s F12 medium, containing 10% heat-inactivated fetal bovine serum (FBS), penicillin (100 U/mL) and streptomycin (100 μg/mL) in a humidified atmosphere with 5% CO_2_ at 37 °C.

#### 3.8.2. In Vitro Growth Inhibition Assay

The assay was performed following the sulforhodamine B (Sigma-Aldrich, St. Louis, MO, USA) method previously published by Villena et al. [47]. Briefly, the cells were set up at 3 × 10^3^ cells per well of a 96-well, flat-bottomed 200 μL microplate. Cells were incubated at 37 °C in a humidified 5% CO_2_/95% air mixture and treated with the extracts at different concentrations for 72 h. At the end of the crude extract exposure, cells were fixed with 50% trichloroacetic acid at 4 °C (TCA final concentration 10%). After washing with distilled water, cells were stained with 0.1% sulforhodamine B, dissolved in 1% acetic acid (50 µL/well) for 30 min and subsequently washed with 1% acetic acid to remove unbound stain. Protein-bound stain was solubilized with 100 µL of 10 mM unbuffered Tris base. The cell density was determined using a fluorescence plate reader (wavelength 540 nm). Untreated cells were used as the negative control while cells treated with doxorubicin and 5-fluorouracil were used as the positive control. In addition, all of the samples were tested from 0 to 100 μg/mL (concentration of extracts) using ethanol as the carrier solvent. Values shown are the mean ± standard deviation of three independent experiments performed in triplicate. Finally, Sigma Plot software was used to calculate the IC_50_ value.

#### 3.8.3. Selectivity Index

The selectivity index (SI) is the quotient of the IC_50_ value of the *P. chilensis* extracts determined for CCD 841 CoN cells and the value obtained for the cancer cell line, and was calculated following Equation (3):

**SI** = IC_50 (CCD 841 CoN)_/IC_50(HT-29 cells)_ (3)

### 3.9. Statistical Analysis

Values are averages ± standard deviation of three independent experiments performed in triplicate. Kruskal–Wallis ANOVA with 95% confidence level using STATISTICA 7.0 was used due to non-parametric data.

## 4. Conclusions

The **EA** extract of the *P. chilensis* flower shows antioxidant activity, outperforming the other extracts in the DPPH, FRAP and TRAP assays, which correlates with the results obtained in this study of its phytoconstituents, since it concentrates the highest amount of phenols, flavonoids and anthraquinones. Among the compounds identified in this extract are mainly fatty acid esters, fatty alcohols, fatty acids and steroids. Some of these compounds, like 2,6-dihexadecanoate ascorbate (15.21%), have antioxidant and antiproliferative activity; 1-tetracosanol (12.57%) has also shown antiproliferative activity, supporting the efficacy of **EA** flower extract in inhibiting the growth of HT-29. In conclusion, the **EA** extract of *P. chilensis* flowers shows a promising profile for antioxidant and antitumor applications, supported by its bioactive compounds. Furthermore, this study demonstrated that its extracts do not cause toxicity in healthy colon cells, which supports its use in traditional medicine. Future research could be carried out to determine the antiproliferative effect of its main compounds separately, to identify the mechanism of action by which they exert their cytotoxic activity.

## Figures and Tables

**Table 1:** Phytoconstituent concentration per extract of *P. chilensis*.

Part of Plant	Extract	Phenols (μg GAE/g d.e.)	Flavonoids (μg QE/g d.e.)	Anthraquinones (μg EE/g d.e.)
**Stem**	**H**	2.63 ± 0.24 ^a^	0.92 ± 0.17 ^a^	1.48 ± 0.12 ^a^
	**EA**	2.66 ± 0.05 ^a^	21.06 ± 0.31 ^b^	0.48 ± 0.20 ^a^
	**E**	2.50 ± 0.14 ^b^	20.29 ± 0.32 ^b^	0.93 ± 0.25 ^a^
**Flower**	**H**	2.84 ± 0.22 ^c^	30.07 ± 0.12 ^c^	14.71 ± 0.38 ^b^
	**EA**	4.63 ± 0.37 ^d^	31.5 ± 0.23 ^c^	12.60 ± 0.20 ^b^
	**E**	2.72 ± 0.32 ^c^	LOD	LOD

Values expressed as the mean values ± standard deviation (*n* = 3). Different letters in the same column indicate significant differences; *p* < 0.05; LOD = limit of detection.

**Table 2:** Antioxidant activity of *P. chilensis* extracts.

Part of Plant	Sample/Extract	DPPH∙ (IC_50_ mg/mL)	FRAP (TEAC mM)	TRAP (TEAC µM)
**Stem**	**H**	91.33 ± 0.26 ^a^	12.53 ± 0.02 ^b^	0.02 ± 0.01 ^a^
	**EA**	44.03 ± 0.21 ^c^	12.61 ± 0.24 ^b^	0.03± 0.01 ^b^
	**E**	11.77 ± 0.17 ^d^	11.80 ± 0.32 ^b^	0.07± 0.05 ^a^
**Flower**	**H**	21.43 ± 0.10 ^e^	12.75 ± 0.01 ^b^	0.03 ± 0.00 ^a^
	**EA**	4.15 ± 0.10 ^d^	26.52 ± 0.01 ^a^	0.47 ± 0.01 ^b^
	**E**	5.32 ± 0.01 ^d^	11.38 ± 0.01 ^b^	0.09 ± 0.01 ^a^
	Trolox	0.26 ± 0.02 ^f^	n.a	n.a
	Gallic acid	0.06 ± 0.01 ^f^	1.72 ± 0.01 ^c^	1.14 ± 0.01 ^c^
	BHT	n.a	1.52 ± 0.07 ^c^	1.06 ± 0.02 ^c^

Values expressed as the mean values ± standard deviation of three independent experiments, each performed in triplicate. Different letters in the same column indicate significant differences; *p* < 0.05; n.a = not applicable.

**Table 3:** Cytotoxic effect of *P. chilensis* extracts (IC_50_ µg/mL) and selectivity index (SI).

Part of Plant	Extract	Cell Lines		SI
		HT-29	CCD 841 CoN	
**Stem**	**H**	>100	>100	I
	**EA**	>100	>100	I
	**E**	>100	>100	I
**Flower**	**H**	>100	>100	I
	**EA**	41.70 ± 0.05	>100	2.40
	**E**	98.6 ± 0.02	>100	1.01
	Doxo	1.75 ± 0.05	5.01 + 0.53	2.86
	5-FU	9.15 ± 0.5	42.41 ± 0.1	4.63

Values expressed as the mean values ± standard deviation of three independent experiments, each performed in triplicate. I= inactive; SI = selectivity index obtained using Equation (3).

**Table 4:** Composition of the volatile fraction of **EA** flower extract from *P. chilensis*.

Nº	RT (min)	Components	%A ^a^	RI ^b^	RI ^c^	Match
1	7.66	2,3-butanediol diacetate	0.17	1075	1080	940
2	8.48	Nonanal	0.09	1106	1102	960
3	8.822	1,3-propanediol diacetate	0.10	1121	RINR	870
4	10.30	benzoic acid	0.05	1186	1191	850
5	10.62	ethyl hydrogen succinate	0.25	1201	RINR	920
6	12.03	benzeneacetic acid	0.28	1279	1276	930
7	12.30	nonanoic acid	0.06	1293	1297	890
8	12.91	2-methoxy-4-vinylphenol	0.02	1328	1330	800
9	13.63	γ-nonanolactone	0.05	1367	1363	910
10	14.34	Vanillin	0.06	1408	1409	870
11	16.91	dodecanoic acid	0.09	1578	1576	860
12	17.10	3-hydroxy-4-methoxybenzoic acid	0.40	1591	RINR	860
13	17.95	3-oxo-α-ionol	0.02	1652	1656	810
14	18.81	ethylhexyl benzoate	0.08	1716	RINR	940
15	19.40	methyl vanillate	0.20	1762	RINR	860
16	19.60	myristic acid	0.33	1776	1775	850
17	19.88	Isophorone	0.30	1798	RINR	810
18	20.83	pentadecanoic acid	0.09	1875	1878	850
19	21.09	*trans*-ferulic acid	0.74	1895	1897	880
20	21.56	hexadecanoic acid methyl ester	5.47	1931	1928	890
21	21.86	9-hexadecenoic acid	2.39	1960	1957	920
22	22.17	ascorbic acid 2,6 dihexadecanoate	15.21	1986	RINR	930
23	22.32	palmitic acid	4.88	1999	1996	900
24	24.17	(*Z*,*Z*)-9,12-octadecadienoic acid	10.41	2083	2095	920
26	26.57	Docosane	0.78	2200	2200	900
27	30.21	Tetracosane	0.39	2400	2400	860
28	30.36	bis(ethylhexyl) sebacate	1.11	2408	RINR	940
29	30.93	1-tetracosanol	9.85	2437	RINR	930
30	32.47	1-pentacosanol	12.57	2529	RINR	930
31	33.06	α-tocopherol	1.77	2563	3112	950
32	34.86	β-sitosterol	2.55	2665	3187	860
33	35.29	20b-Dihydroprogesterone	0.92	2690	RINR	800
34	35.73	9,19-cyclolanost-24-en-3-ol	0.69	2715	3465	850
35	36.53	stigmast-4-en-3-one	10.59	2761	3458	880
		Known compounds	82.96			
		Unknown compounds	17.04			

^a^ Surface area of GC peak; ^b^ experimental retention index for RTX-5 capillary column; ^c^ bibliographic retention index [32]; RINR = retention index not reported on column with similar polarity.

## Data Availability

The original contributions presented in this study are included in this article; further inquiries can be directed to the corresponding authors.
